# Influence of subway train fire locations on the characteristics of smoke movement in a curved tunnel

**DOI:** 10.1371/journal.pone.0279818

**Published:** 2023-01-03

**Authors:** Dan Zhou, Jinzhu Li, Tianen Hu, Tao Chen

**Affiliations:** 1 Key Laboratory of Traffic Safety on Track, Ministry of Education, Central South University, Changsha, China; 2 Joint International Research Laboratory of Key Technology for Rail Traffic Safety, Changsha, China; 3 National & Local Joint Engineering Research Centre of Safety Technology for Rail Car, Changsha, China; Southwest Jiaotong University, CHINA

## Abstract

Scenario models of a moving subway train can help investigate the influence of different fire locations on smoke propagation characteristics in curved tunnels. To this end, this study adopts the three-dimensional Unsteady Reynolds Average Navier-Stokes equations method and the renormalization group *k-ε* two-equation turbulence model with buoyancy correction for numerical analysis. The motion of the train is replicated using the slip grid technique. The results indicate that when a fire breaks out on a moving train in tunnels, the piston wind leads the longitudinal movement of the smoke. If a fire erupts in the head or middle car of a moving train, the time of smoke backflow is delayed by 30 s or 17 s, respectively, compared to that for the tail car. The obtained results provide a theoretical basis for reasonably controlling the smoke flow in subway tunnels and reducing casualties in fire accidents.

## 1. Introduction

Urban subways are characterized by a high ridership, safety and reliability; furthermore, they use fewer surface transportation resources. Although subways provide convenience to people, safety problems are becoming more apparent. Fires on subway trains are among the most serious disasters, causing casualties and property damage. For example, the Daegu subway fire in South Korea in 2003 claimed 192 lives and injured 148 people [1]; moreover, the London subway fire in the United Kingdom in 2005 claimed 52 lives and injured more than 700 people [2]. Given the incalculable losses caused by fires in tunnel, many scientists have carried out a number of studies on the characteristics of smoke propagation in tunnels.

Due to the topography and location of stations, many subway tunnels have some curvature. Limited by research objects, experimental conditions, and other factors, many researchers have used stationary fire sources to study the diffusion law of fire smoke in tunnel. Wang et al. [3] investigated the law of critical ventilation velocity in a curved tunnel by placing a fire source at different lateral positions in the tunnel and investigated the length of smoke counterflow of the fire source from different lateral positions. In this regard, Lou et al. [4] investigated the smoke flow in tunnels with different curvatures under multiple heat source conditions. They reported that the temperature and velocity of smoke were higher on the outside of the tunnel near the fire source than on the inside of the tunnel; however, this difference decreased as the radius of the curve increased. Wang et al. [5] studied the effects of tunnel slope and curvature on smoke flow under natural ventilation conditions. Zhang et al. [6] studied the effects of different heat source powers on critical ventilation velocity in a curved subway tunnel with different radii. Zhong et al. [7] conducted large-scale experiments to study the smoke propagation characteristics of three different fire sources in a curved tunnel. Caliendo et al. [8] built a numerical simulation model for a heavy truck fire in a curved bi-directional tunnel and investigated the effects of truck location, tunnel geometry, and longitudinal ventilation velocity on smoke concentration and on visibility distance with regards to the human eye.

When a train catches fire while moving, it tends to continue for a certain distance. Zhong et al. [9] reported that the resulting fire smoke creates a large vortex and a strong horizontal inertial force under the action of the piston wind of the train. Some researchers have used three-dimensional simulation models to study the effects of longitudinal ventilation speed and train/tunnel blockage ratio on smoke dispersion when a subway train catches fire and is forced to reduce its speed to stop in a tunnel [10, 11]. Bai et al. [12] created a tunnel model with a rescue station and a cross passage as well as studied the flow field in the tunnel when the combustive train approached the rescue station. The results demonstrate that the change in the flow field during the emergency braking of a train has an obvious effect on the diffusion of smoke in the rescue station. Wang et al. [13] used a moving model experiment to investigate the impacts of train speed and blocking ratio on the features of smoke movement in a tunnel. Zhou et al. [14] reported that tunnel slope significantly affects the temperature distribution and the time of smoke backflow in the tunnel with a moving subway train on fire.

Currently, research on fires in curved subway tunnels mainly focus on stationary fire sources. Some researchers have also studied fires in moving subway trains, but the fire scenarios mainly focus on straight tunnels. Some studies have reported that the lateral locations of fire source from the tunnel wall affect the critical ventilation velocity and longitudinal temperature field, but these studies ignore the influence of the train blocking ratio [15, 16]. According to the results of fire research in tunnel, the blocking ratio between the tunnel and train critically impacts smoke diffusion [17, 18]. The blocking lengths upstream and downstream of the fire source depend on the position where the train catches fire. However, the influence of the location of the train fire on smoke propagation is still unclear.

In this research, the scenario of a moving subway train on fire in a curved tunnel is simulated. Moreover, smoke dispersion characteristics are investigated with regards to the fire source locations on a subway train. The distribution characteristics of smoke movement under different fire scenarios are also determined. The study results can provide a theoretical basis for reasonably controlling the smoke flow in subway tunnels and for reducing casualties in fire accidents.

This paper is generally divided into four sections. The first section mainly introduces the background of this research. The second section briefly explains the geometric model and the numerical calculation method used in this study. The third section discusses the influence of the locations of subway train fire on the characteristics of smoke movement in a curved tunnel. The fourth section generalizes the conclusions obtained in this study and establishes the scope for possible future research.

## 2. Numerical analysis

### 2.1. Computational model

**Fig 1:**
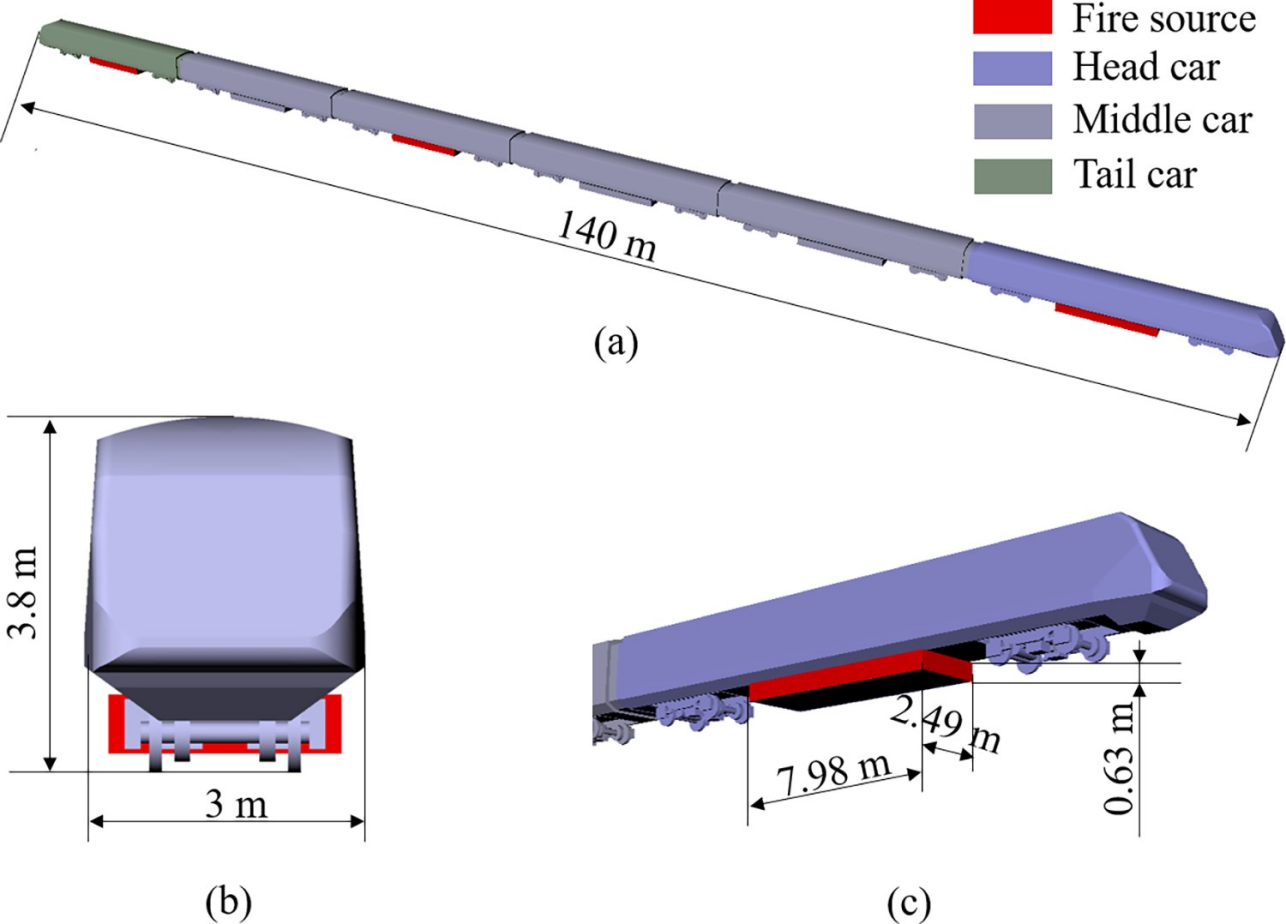
Geometric model of the train. (a) 3D view; (b) right side view; (c) diagram of equipment cabin.

This study considers a six-group subway train as the research object. A geometric model of the train is displayed in Fig 1. The geometric parameters of the train are 140 m long, 3 m wide, and 3.8 m high, with an area of cross-section of 9.5 m^2^. The train equipment cabin is a cuboidal structure with a length, breadth, and height of 7.98 m, 2.49 m, and 0.63 m, respectively. For simulating a tunnel train fire, the train model includes detailed structures, such as the equipment cabin and the bogie of the subway train, which can reduce the impact of model accuracy on simulation results by refining train modeling.

**Fig 2:**
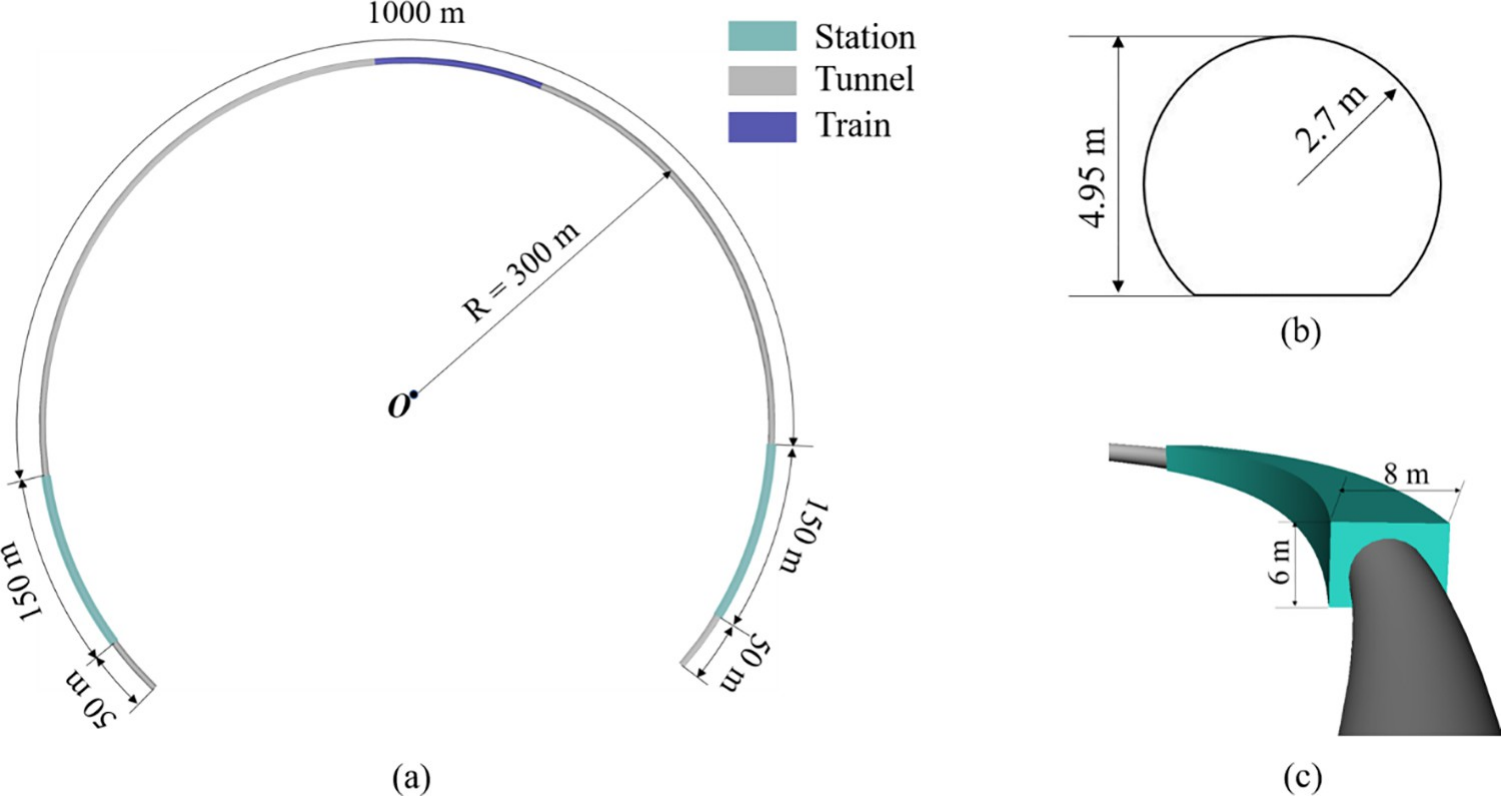
Geometric model of the tunnel. (a) Overall top view; (b) section of tunnel; (c) station model.

Fig 2 depicts the model of the curving tunnel. The middle tunnel is 1000 m long and has an area of cross-section of 22.4 m^2^. The dimensions of the platforms are 150 m long, 8 m wide, and 6 m high. The tunnels at the outer ends of the two platforms are extended by 50 m. The "Code for Design of Metro" states that the minimum curve radius of the A-type metro running line should not be less than 250 m [19]. After considering multiple practical application cases, a tunnel curve radius of 300 m is selected for further analysis.

### 2.2. Methodology for fire source numerical simulation

#### 2.2.1. Turbulence model

Numerical simulation calculations can use various methods to study problems related to trains/tunnels, including 1D/2D/3D, compressible/incompressible, and steady/unsteady flow methods [20–23]. The numerical calculation methods used for different research problems are often different.

The propagation of fire smoke in subway tunnels is a turbulent motion that occurs under the combined effects of a train slipstream and thermal buoyancy. In this study, the Unsteady Reynolds Average Navier-Stokes equations (URANS) method is used to deal with the Navier-Stokes equations, which reduces the computational complexity of the simulation process while ensuring its accuracy [10]. For the multi-component fluid that includes train fire smoke, the SIMPLE algorithm is employed to solve the pressure spatial discretization. The “Body Force Weighted” approach is also used to improve the accuracy of the simulation. Simultaneously, the RNG *k-ε* model with buoyancy correction is used to improve the accuracy of the fire smoke dispersion process simulation [24]. In addition, the buoyancy-driven flow is simulated by applying the Boussinesq model. The equations of RNG *k-ε* turbulence model are as follows.

Turbulent kinetic energy *k* equation:

$$\frac{\partial }{\partial t}(\rho k)+\frac{\partial }{\partial x_{i}}(\rho u_{i}k)=\frac{\partial }{\partial x_{j}}(\alpha_{k}\mu_{eff}\frac{\partial k}{\partial x_{i}})+G_{k}+G_{b}-\rho \varepsilon -Y_{M}+S_{k}$$ (1)

Turbulent dissipation rate *ε* equation:

$$\frac{\partial }{\partial t}(\rho \varepsilon )+\frac{\partial }{\partial x_{i}}(\rho u_{i}\varepsilon )=\frac{\partial }{\partial x_{j}}(\alpha_{\varepsilon }\mu_{eff}\frac{\partial \varepsilon }{\partial x_{j}})+C_{1\varepsilon }\frac{\varepsilon }{k}(G_{k}+C_{3\varepsilon }G_{b})-C_{2\varepsilon }\rho \frac{\varepsilon^{2}}{k}-R_{\varepsilon }+S_{\varepsilon }$$ (2)

The finite volume method used in this study is a numerical methodology commonly used in numerical simulations. The computational domain is solved using the commercial general-purpose software Fluent 18.1. The software Fluent 18.1 has rich physical models and advanced numerical methods as well as powerful pre-processing and post-processing functions; moreover, it is widely applied in the fields of fluids, heat transfer, and chemical reactions.

#### 2.2.2. Radiation model

Radiation models are used to simulate the radiative heat transfer between heat sources, fluids, and solid walls during fires. The length of the tunnel in this study is 1000 m, and the fire smoke spreads along the tunnel widely, which is suitable for the Rosseland radiation model [10]. For the fires studied in this paper, the optical depth is far greater than 3. Moreover, the Rosseland radiation model does not calculate the transport equation of medium radiation intensity. Therefore, Rosseland radiation model is beneficial for accurately simulating fire in tunnel and speeding up the calculation progress. The radiation flux *Q*_*r*_ of the Rosseland model is obtained by a theoretical formula [24].

#### 2.2.3. Combustion model

In this study, the most commonly used volumetric heat source model in engineering simulation is used [25–27]. The model ignores the authentic chemical reactions in the fire combustion process but simulates the specific heat release and smoke evolution rates. A steady-state volume heat source with a constant fire source power is used to simulate the heat release and smoke products generation when train fires occur in railway tunnels. This approach has been also supported by many other researchers [28, 29].

(1) Fire source power and smoke release rate.

The extent of fire in subway trains depends on various factors, and the power range is generally 5 MW to 10.5 MW [19]. After analyzing the relevant equipment and safety standards of the selected subway train, the power of the fire source is determined as 7.5 MW to simulate train fires at different locations. The smoke released from the combustion of the fire source is determined as carbon dioxide; moreover, the total smoke emission and the smoke type are expressed by one smoke type. The smoke release rate is calculated using the following formula [30]:

$$m_{c}=0.071Q_{c}^{\frac{1}{3}}Z^{\frac{5}{3}}+0.00192Q_{c}$$ (3)

where *Q*_*c*_ is usually 0.7 times the power of *Q*. The smoke release rate is related to the height of the smoke relative to the tunnel floor. Therefore, the maximum smoke release rate for a fire power of 7.5 MW in the tunnel is 27.78 kg/s.

(2) Locations of the fire source

Train equipment compartments usually contain electrical equipment, such as traction transformers, traction converters, and auxiliary power supply units. According to the statistics of train fire accident, the failure of train electrical equipment is the leading cause of fires [31]. Compared with the scenario of fire inside the train, the fire at the bottom develops faster under the action of the piston wind, further increasing the danger. Therefore, in this study, "train fire" means models are established for fires in the equipment compartments of the train head, middle (fourth car), and tail cars.

### 2.3. Computational grid and boundary conditions

**Fig 3:**
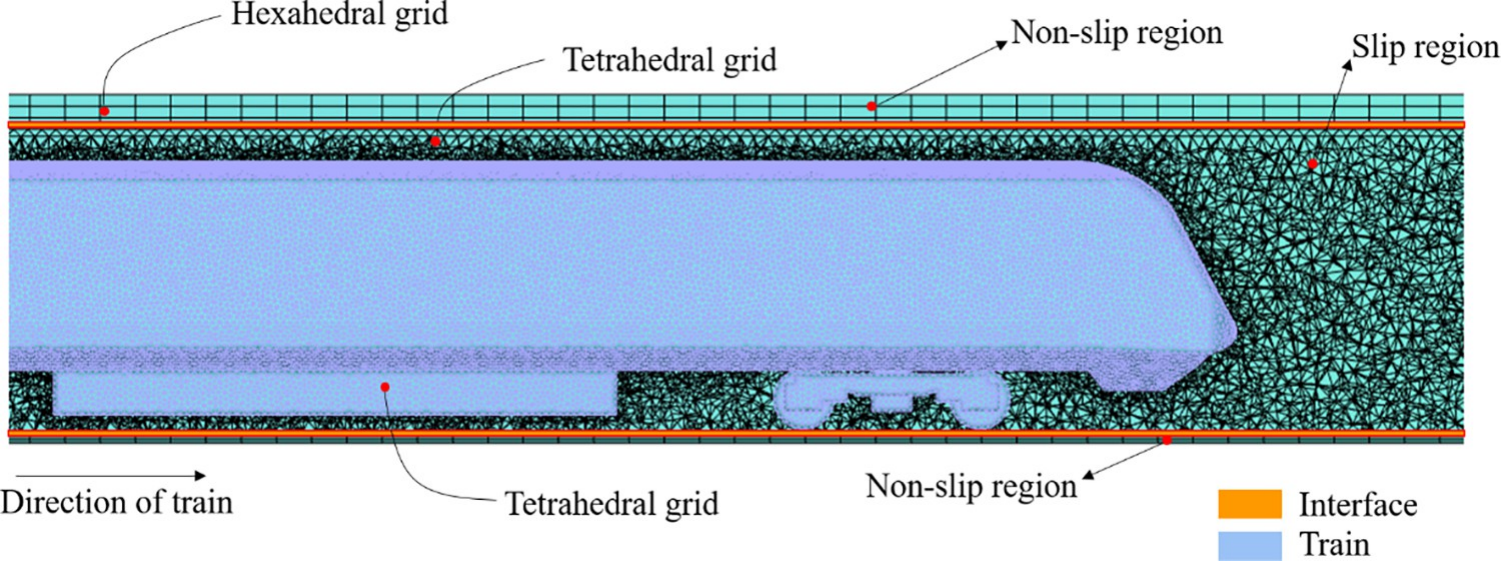
Local grids in the computational domain.

Considering the influence of train blockages on smoke propagation, a refined subway train model is adopted. The local mesh is shown in Fig 3. The train model includes the windshield, bogie, and some other components. Due to the complex surface structure of the train, an unstructured grid with high adaptability is used to subdivide it. The grid around the fire source is refined, and the grid size is in the range of 0.08 m to 0.10 m to ensure that the numerical solution of the flow field is more accurate. Furthermore, the non-slip region is divided into structured grids. The number of grids in the entire computational domain is 8.6 million.

**Fig 4:**
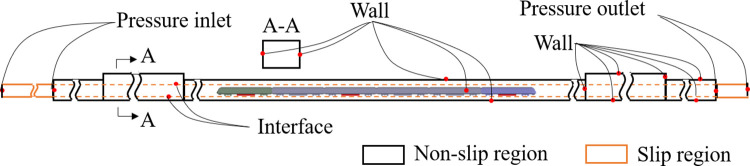
Computational domain and boundary conditions.

The boundary conditions of the developed model are shown in Fig 4. The boundary conditions at both end sections of the tunnel are defined as the pressure outlet and inlet, respectively. The train, platform, and other tunnel surfaces are set as non-slip walls. The top surface of the fire source is the wall surface, and the other surfaces are defined as the internal surfaces. Considering the influence of the tunnel wall and train surface on temperature change when a fire breaks out, the wall materials of the tunnel and train are specified as concrete and aluminum, respectively. Additionally, their corresponding densities are 2200 kg/m^3^ and 7850 kg/m^3^; thermal conductivities are 1.2 W/(m·K) and 3 W/(m·K); and specific heat capacities are 0.88 kJ/(kg·K) and 0.50 kJ/(kg·K). The initial environmental conditions of the entire computational domain are set as follow: the ambient temperature is 300 K, and the pressure is 101 kPa [32].

### 2.4. Arrangement of measuring points

**Fig 5:**
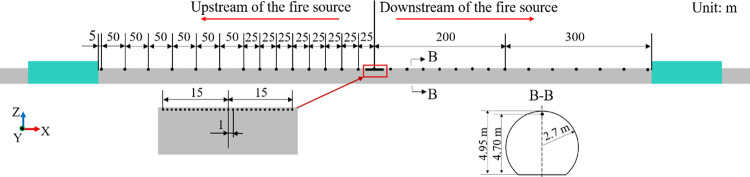
Schematic of the measurement points arrangement.

To monitor the characteristic parameters of the smoke in the tunnel after the train catches fire, some measurement points are established inside the tunnel. The arrangement of measurement points is shown in Fig 5. The final parking positions of the stationary and moving fire sources are the centers of the tunnel. Therefore, the longitudinal midpoint of the tunnel is set as the center, and a measurement point is established every 25 m within 200 m of the center. Outside this area, a measurement point is set every 50 m. Due to the large rate of change in smoke temperature and concentration near the fire source, a measurement point is established every 1 m within 15 m of the tunnel center. All measurement points are placed on the vertical plane of symmetry of the tunnel, and the distances from the ground are 4.7 m. The total number of measurement points are 57.

### 2.5. Mesh independence and model test validation

In this study, a moving model of train on fire is used to verify the numerical calculation method [13]. The moving model is tested to simulate the relative motion of the train and tunnel. Note that when the train is moving with fire source, the slipstream dominates the movement of fire smoke in the tunnel; however, thermal buoyancy has a limited influence. The Reynolds number similarity criterion is mainly guaranteed in the moving model test. According to the criterion of critical Reynold’s number, the flow field around the train in the scale model test is similar to that in the full-scale test. Therefore, it can be used to study the influence of slipstream on smoke movement characteristics for a moving train on fire.

**Fig 6:**
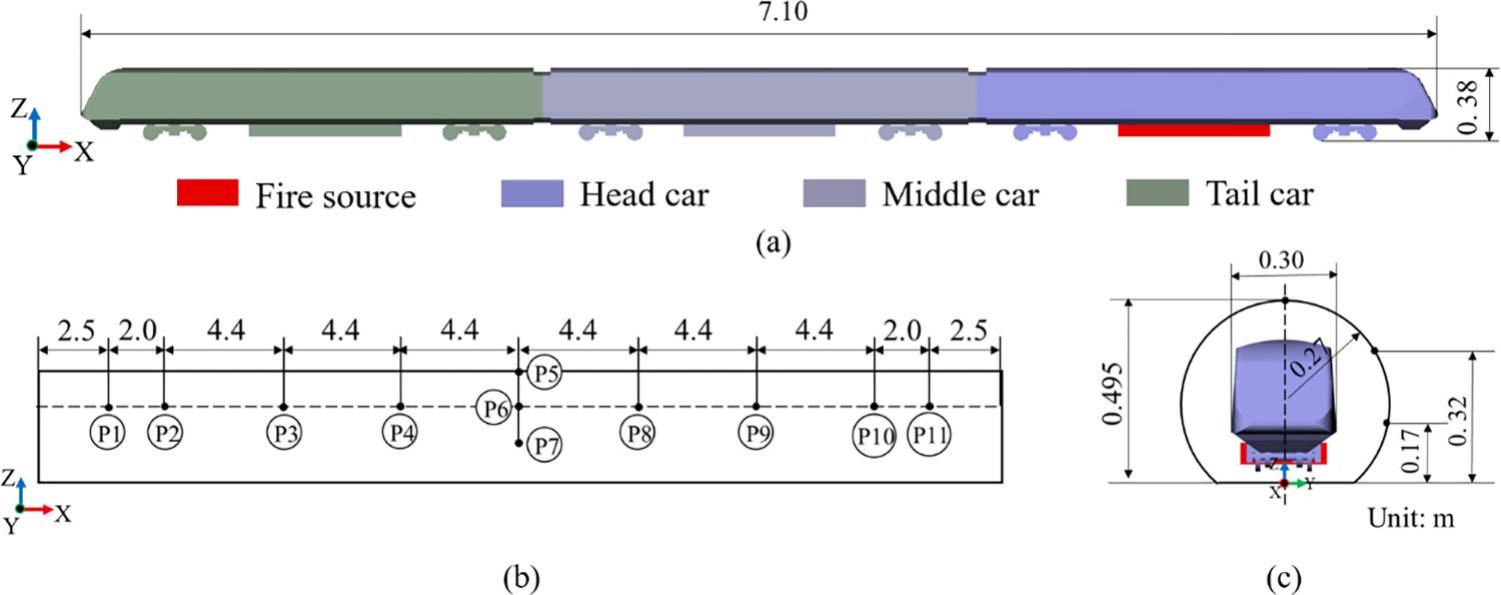
Train and tunnel model diagram with the arrangement of measurement points in the test.

As described in Fig 6, a three-car marshalling model is used for the test. Both the train and tunnel models have a scale of 1:10. The length, width, and height of the train model used in the test are 7.1 m, 0.313 m, and 0.367 m, respectively. Furthermore, the tunnel model has a cross-sectional area of 0.22 m^2^, and the blocking ratio of the train to tunnel is 0.43. The speed of the model train is 60 km/h during the test.

For verifying the numerical calculation method, a geometric model of the same size as that used in the moving model test is adopted. The accuracy of the numerical calculation method is verified by the airflow velocity and the peak smoke concentration parameters at the measurement points in the experiment. In the experiment, the smoke concentration was measured by the concentration sensors, and in the simulation software the measurement points automatically collected the information of smoke concentration in the tunnel. The locations of all measurement points in the test are displayed in Fig 6. To ensure that the meshing method does not affect the numeral calculations results, three schemes of mesh scale are designed simultaneously for verification. The scales of grid among different grid schemes are set to a growth rate of approximately 1.2. Simultaneously, the numerical simulation results of different grid scale schemes are compared with the results from the moving model test. The grid parameters are listed in Table 1. By comparing the results of three mesh schemes, the errors of smoke velocity and smoke concentration between the coarse-mesh scheme and medium-mesh scheme are 6.2% and 4.5%, respectively. Meanwhile, the errors of smoke velocity and smoke concentration between medium-mesh and fine-mesh schemes are 3.1% and 0.8%, respectively. The errors of the medium- and fine-mesh scale schemes is smaller than that of the coarse- and medium-mesh scale schemes. Therefore, the mesh independence verification results are acceptable.

**Table 1:** Parameter values for verifying mesh independence.

Mesh	Minimum grid size (m)		Longitudinal size of grid in the tunnel (m)	Total grids (10^6^)
	Train surface	Tunnel surface		
Coarse	0.010~0.012	0.03	0.08	6.2
Medium	0.008~0.010	0.02	0.06	8.6
Fine	0.006~0.008	0.02	0.04	12.0

**Fig 7:**
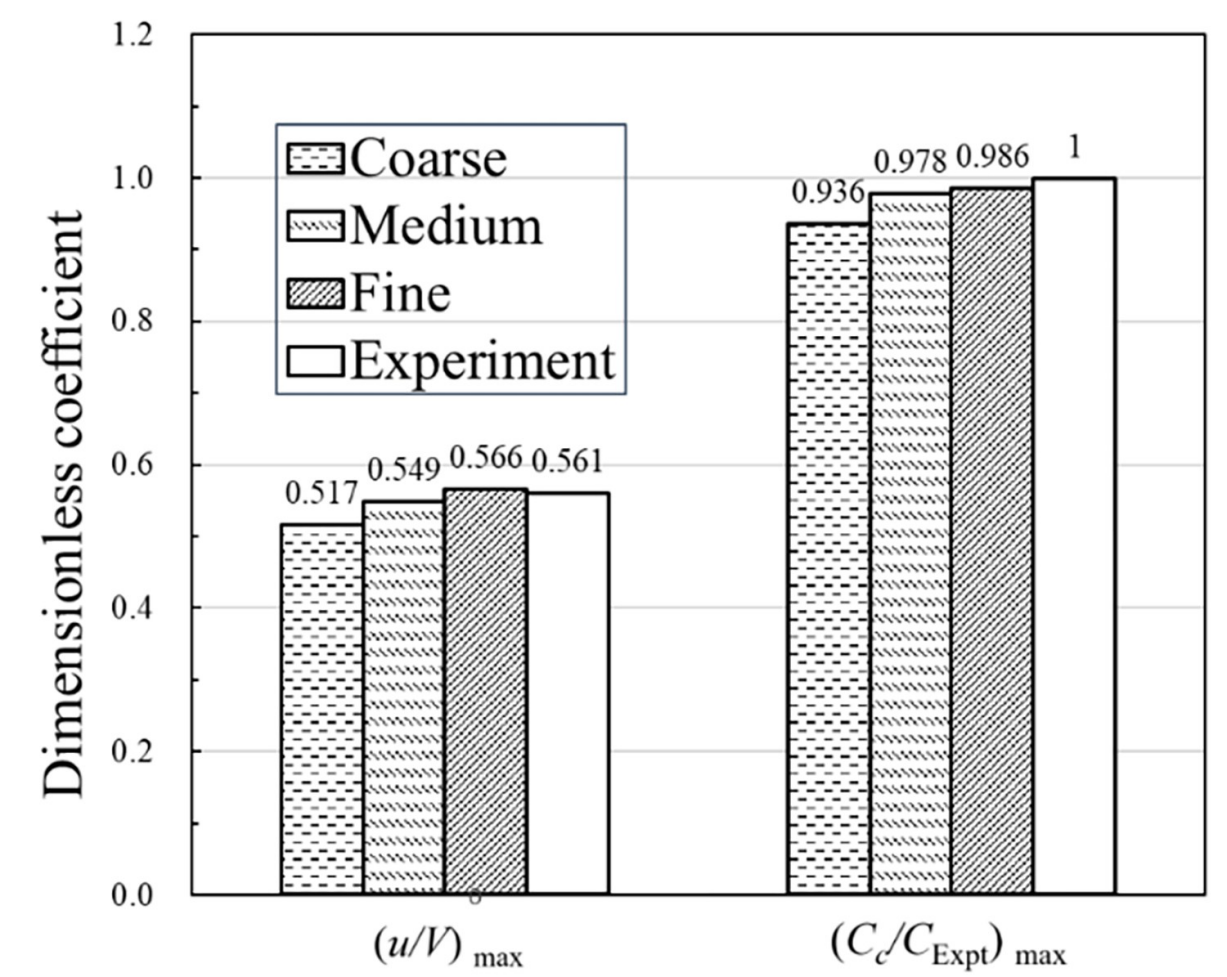
Validation of the results from the numerical calculation method.

At the same time, in order to verify the reliability of the numerical simulation method, a comparison between the numerical simulation results of different mesh scale schemes and the moving model test results are shown in Fig 7 and Table 2. The (*u/V*)_max_ parameter represents the maximum value of the dimensionless flow velocity at P6 measurement point. *C*_c_ represents the smoke concentration at P3 measurement point in the numerical simulation, and *C*_Expt_ represents the maximum value of smoke concentration at P3 measurement point in the moving model experiment. (*C*_c_*/C*_Expt_)_max_ represents the maximum ratio of dimensionless smoke concentration at P3 measurement point. By comparing the peak values of dimensionless flow velocity and smoke concentration, the numerical simulation results of the medium- and fine-mesh scale schemes are verifiably in a good agreement with the results of the moving model test. In contrast, a relatively large error exists between the results of coarse-mesh scheme and the moving model test.

**Table 2:** Comparison of the experimental and numerical calculation methods.

Mesh	Total grids (10^6^)	Smoke velocity		Smoke concentration	
		(*u/V*) _max_	Diff./Expt.	(*C*_c_*/C*_Expt_) _max_	Diff./Expt.
Experiment	/	0.561	/	1	/
Coarse	6.2	0.517	-7.84%	0.936	-6.40%
Medium	8.6	0.549	-2.14%	0.978	-2.20%
Fine	12.0	0.566	0.89%	0.986	-1.40%

From Table 2, the error in the numerical simulation results under the coarse mesh scale is evidently greater than 5% compared with the moving model results. However, the simulation calculation errors from the medium- and fine-mesh scale schemes are all within 5%. Therefore, the numerical simulation with the medium- and fine-mesh scale schemes can accurately reproduce the real flow field. Due to computational cost and time constraints, the medium-mesh scheme in Table 2 is selected for the subsequent numerical simulation analysis.

## 3. Results and discussion

This section examines the distribution of smoke velocity, temperature, and concentration when a moving subway train catches fire in a tunnel with a curve radius of 300 m. The power of the fire is 7.5 MW in the equipment compartments of the head, tail, and middle cars (fourth car) of the train.

**Fig 8:**
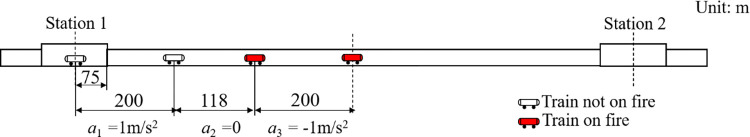
Scenario of a moving fire source.

**Fig 9:**
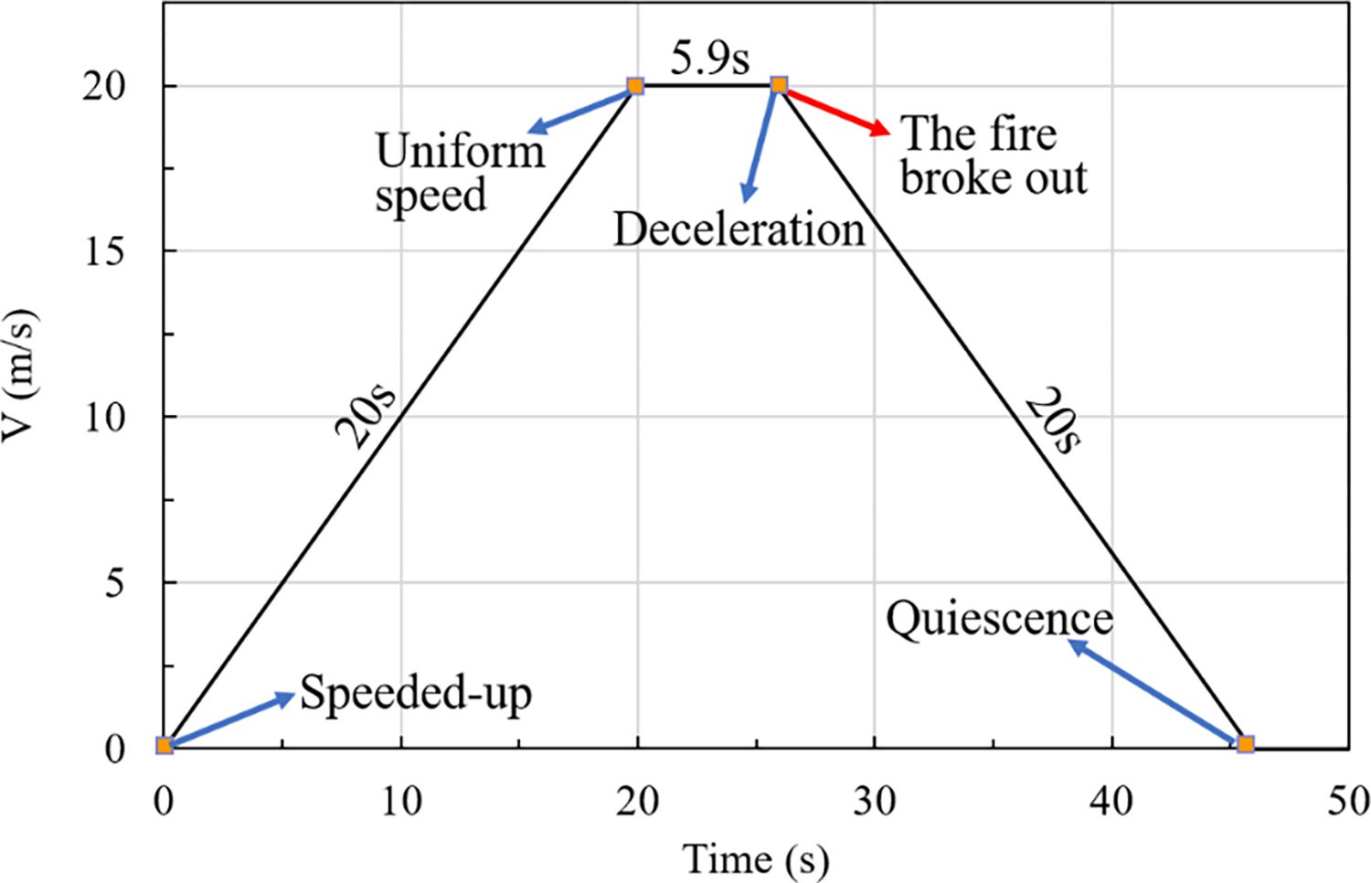
Curve of train speed.

The movement scenario is displayed in Figs 8 and 9. At the beginning, the train leaves station 1 with an acceleration of 1 m/s^2^ from a stationary state. After a travel time of 20 s, the normal operating speed of the train is 20 m/s. Next, the train travels with a constant speed of 20 m/s for 5.9 s and then a fire breaks out. The train then immediately decelerates at a rate of 1 m/s^2^ and finally stops in the middle of the tunnel after 20 s. (Note that the time at which the train starts moving is *t* = 0 s; the center of fire source is the origin of the coordinates, and the positive direction of the x-coordinate points to the travel direction of the train.)

### 3.1. Smoke velocity in the tunnel

**Fig 10:**
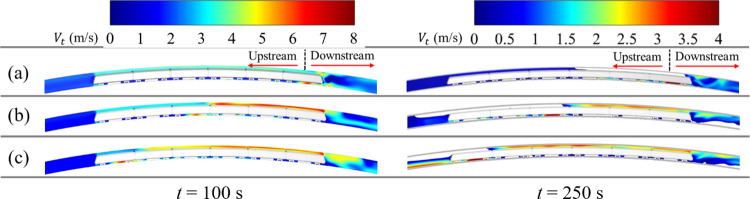
Smoke velocity for different fire sources. (a) Head car; (b) middle car; (c) tail car.

Fig 10 shows the distribution of longitudinal smoke velocity at 100 s and 250 s when the train fire source is at different locations. In Fig 10, the white regions around the train indicate that the smoke velocity is less than zero. As can be seen from Fig 10, the closer the source of the fire is to the tail car when the smoke is not yet flowing in the reverse direction, the higher the smoke velocity in the area where the train is blocked.

**Fig 11:**
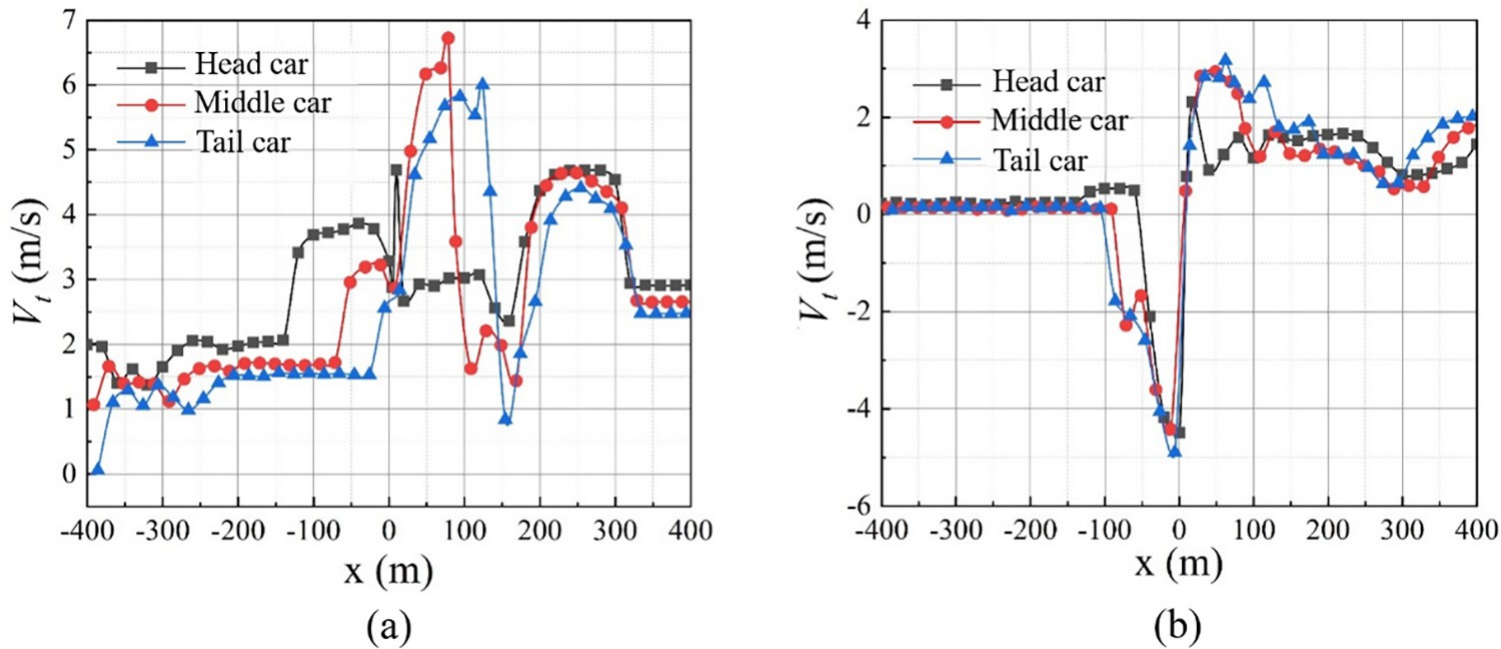
Distribution of smoke velocity for different fire locations. (a) *t* = 100 s; (b) *t* = 250 s.

In Fig 10, the smoke upstream of the fire source exhibits a reverse flow phenomenon when *t* = 250 s. In conjunction with the longitudinal airflow velocity distribution of the tunnel vault at different fire sources (see Fig 11), note that the closer the fire is to the head car, the shorter the length of the backflow of smoke and the smaller the peak value of the backflow velocity. When the head car is on fire, the length of the smoke backflow is 55 m, and the peak value of the backflow velocity is 4.50 m/s. In the case of a tail car fire, the length of the backflow of smoke is 104 m, and the peak value of the backflow velocity is 4.89 m/s; these values represent an increase of 89.09% and 8.67%, respectively, over the scenario of head car fire.

Table 3 lists the times at which the smoke flows backward in the tunnel at various fire locations. Compared with the scenario of tail car fire, the time for the backflow of smoke is delayed by 30 s or 17 s when the head or middle car is on fire, respectively.

**Table 3:** Time of smoke counterflow for different fire sources.

Fire location	Time of smoke counterflow
Head car	169 s
Middle car	152 s
Tail car	139 s

### 3.2. Temperature in the tunnel

**Fig 12:**
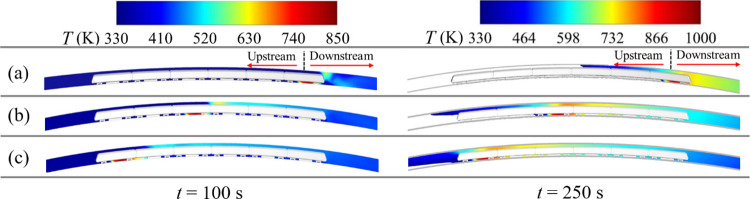
Temperature variation for different fire locations. (a) Head car; (b) middle car; (c) tail car.

Fig 12 indicates the longitudinal temperature distribution at *t* = 100 s and 250 s when the fire source is at different locations. In Fig 12, the white regions around the train mean that the temperature in the tunnel is less than 330 K. Note that at *t* = 100 s, no counterflow of smoke exists upstream of the fire source.

**Fig 13:**
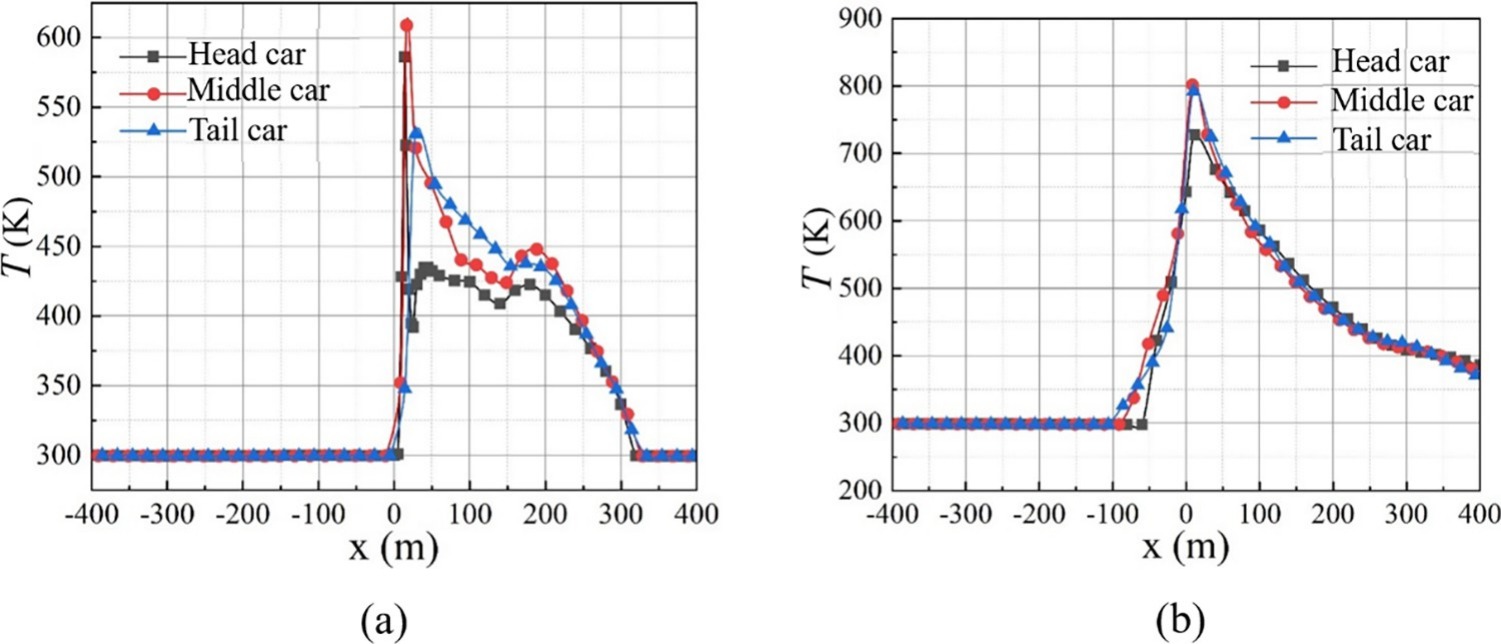
Distribution of temperature for different fire locations. (a) *t* = 100 s; (b) *t* = 250 s.

Fig 13A displays the longitudinal temperature distribution of tunnel vault at *t* = 100 s. When the tail car is on fire, the temperature peak at the tunnel vault is the smallest, but the vault temperature is the highest downstream from 30 m to 150 m, compared with the scenarios of head and middle car fires. When the head car is burning, the peak temperature of the tunnel vault is 55.24 K higher than when the tail car is burning. This is because the train-blocking effect is not significant downstream. Consequently, the smoke velocity decreases near the fire source, and the smoke spreads slowly downstream.

When the tail car catches fire, the tunnel temperature peak at 250 s increases from 530.66 K to 792.50 K, an increase of 49.34% compared to the time of 100 s. However, during this 150 s period when the head car is burning, the peak temperature only increases by 24%. This can be explained by the fact that when a fire occurs in the tail car, the smoke velocity downstream is high, causing the air near the fire to mix well with hot smoke.

Table 4 lists the peak values of temperature and dispersion distance of smoke upstream and downstream at *t* = 250 s for different fire locations. The dispersion distance of the smoke for a fire in the tail car is 83 m, which is 56.60% greater than that for the head car fire. However, the downstream distance of the smoke is 3.40% smaller than that for the head car fire. To summarize, the closer the fire occurs to the tail car, the longer the upstream distance of the smoke and the shorter the downstream distance of the smoke.

**Table 4:** Temperatures for different fire locations at *t* = 250 s.

Fire location	Temperature peak (K)	Smoke diffusion length (m)		
		upstream		downstream
Head car	726.55	53	500	
Middle car	802.09	74	500	
Tail car	792.50	83	483	

### 3.3. Smoke concentration in the tunnel

After the fire starts, a large amount of smoke spreads rapidly, resulting in a sharp increase in smoke concentration. Smoke concentration is defined as the proportion of smoke mass per unit mass of air (C). When the smoke concentration exceeds 0.03, people experience dizziness, nausea, and other reactions, severely limiting their ability to escape. If the smoke concentration continues to increase, suffocation can occur. Therefore, smoke is said to be highly concentrated when the smoke concentration (C) is greater than 0.03.

**Fig 14:**
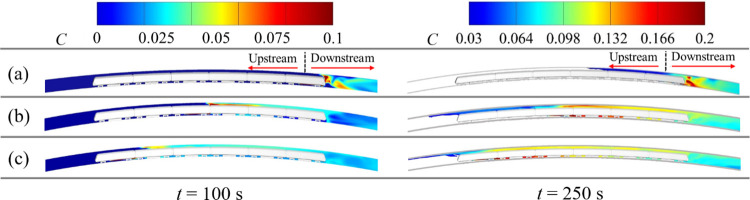
Smoke concentrations for different fire locations. (a) Head car; (b) middle car; (c) tail car.

Fig 14 shows the longitudinal distribution of smoke concentration around the train at *t* = 100 s and 250 s when the fire source is at different locations. In Fig 14, the white regions around the train mean that the smoke concentration in the tunnel is less than 0.03. Fig 14 demonstrates that a high smoke concentration is distributed mainly in the ceiling of the tunnel when a fire starts in the tail or middle car. When the head car is on fire, the high-concentration smoke spreads mainly in front of the window of driver’s room and on the floor of the tunnel. Before the smoke countercurrent, the smoke spreads along the tunnel vault to the tunnel exit. After the smoke is refluxed, it spreads to the tunnel exit and entrance.

In addition, the total amount of fresh air in the blocked area of the train is less than that in the unblocked area. When the fire source produces the same amount of smoke, the smoke concentration increases in the blocked area. Therefore, the peak smoke concentration is lowest when the head car is on fire. The results are shown in the distribution of smoke concentration in the tunnel vault (see Fig 14).

**Fig 15:**
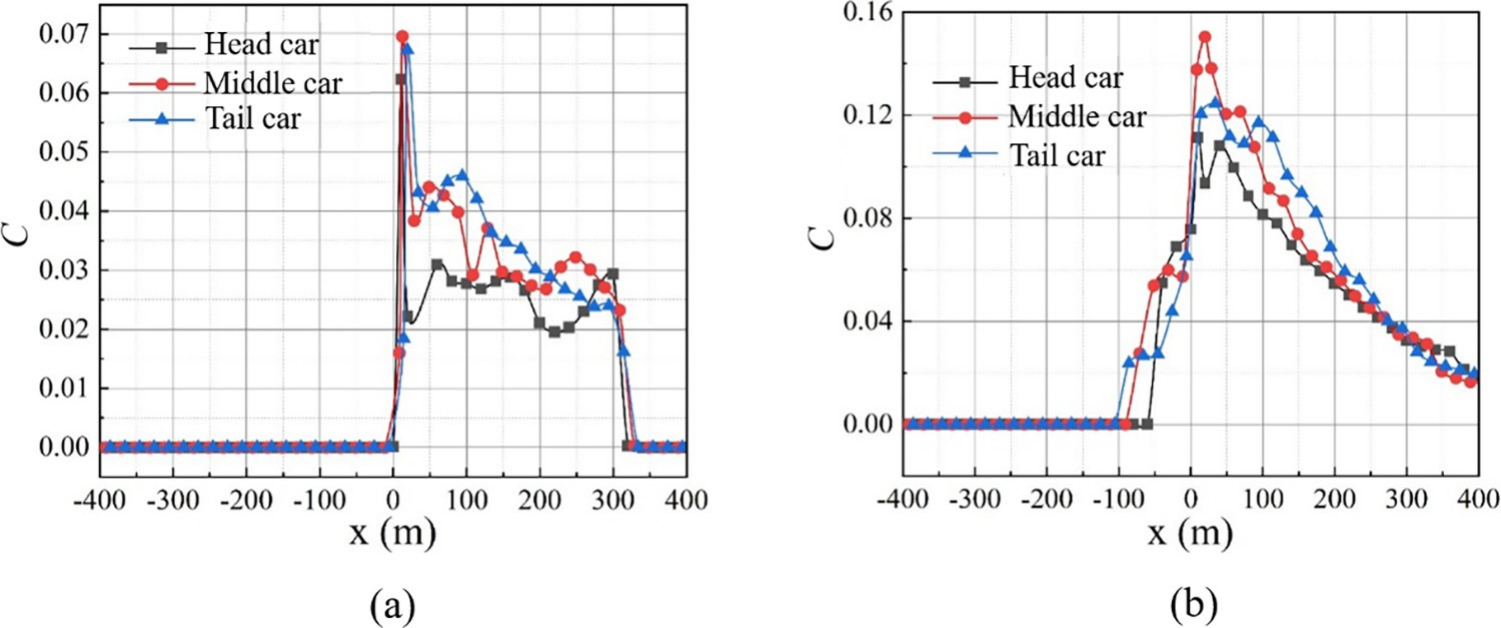
Distribution of smoke concentration for different fire locations. (a) *t* = 100 s; (b) *t* = 250 s.

When the middle car catches fire, the peak of smoke concentration is always higher than that when the tail car catches fire. After the smoke countercurrent occurs, the difference between them increases further. Compared with Fig 15, the difference for peak smoke concentrations in the tunnel between the middle and tail cars increases from 0.0023 at 100 s to 0.025 at 250 s. When the smoke refluxes (see Fig 15B), the upstream blockage length of the fire source is longer for the middle car than that for the tail car fire, which means that fresh air is less in the blocked space. Therefore, assuming that the same amount of smoke is released from the fire, the smoke concentration is higher when the middle car is burning. When the tail car catches fire, more high-temperature smoke spreads to the space behind the train, reducing the high smoke concentration.

Table 5 lists the peak values of smoke concentration and the diffusion distances of highly concentrated smoke at *t* = 250 s for different fire locations. Furthermore, the diffusion distance of highly concentrated smoke upstream is the longest when the middle car is burning. It is 30.19% and 60.47% longer than that for the head and tail car fires, respectively.

**Table 5:** Smoke concentration for different fire locations at *t* = 250 s.

Fire location	Peak of smoke concentration	Peak position (m)	Diffusion range of high-concentration smoke(m)	
			upstream	downstream
Head car	0.111	+7	53	320
Middle car	0.150	+20	69	305
Tail car	0.125	+37	43	306

## 4. Conclusion

During this research, a numerical methodology is adopted to investigate entire fire history in a curved tunnel with a moving subway train on fire. The influence of different fire locations on the spread of smoke flow velocity, temperature, and concentration is analyzed. The key findings are listed below:

1. For a moving train with fire in a tunnel, the smoke propagation was affected by the moving air in the slipstream of the train and spread rapidly along the direction of the train. The distribution characteristics of smoke were complicated.
2. When a fire breaks out on a moving train, the closer the fire is to the tail car, the longer the length of the countercurrent and the peak countercurrent velocity are. Simultaneously, the upstream diffusion distance of the highly concentrated smoke is larger. Compared with the scenario of tail car fire, the timing of smoke backflow is delayed by 30 s or 17 s when the head or middle car is burning, respectively.
3. The location of the fire determines the blockage length of the train/tunnel upstream and downstream of the fire source, which substantially impacts the smoke dispersion of a moving train on fire.

Due to the limitation of conditions, the research adopts some idealized models. The current work still needs further research in the following aspect: This paper only studies the condition of a single curve tunnel, and following research can be carried out in combination with other curved radius tunnels to form a systematic research theory.

## Abbreviations

*C*: smoke concentration
*C_1ε_*: constant in Eq (2)
*C_2ε_*: constant in Eq (2)
*C_3ε_*: constant in Eq (2)
*C_ref_*: highest smoke concentration measured throughout the experiment
*G_b_*: generation due to the mean velocity gradients in Eq (1)
*G_k_*: generation due to buoyancy in Eq (1)
*k*: turbulence kinetic energy (J)
*m_c_*: mass flow rate of smoke (kg/s)
*Q*: fire source power (MW)
*Q_c_*: convective heat flux (kW)
*Q_r_*: radiation flux (kW)
RNG: renormalization group
*R_ε_*: additional term in the ε equation
*S_k_*: user-defined source term
*S_ε_*: user-defined source term
*t*: time (s)
*T*: temperature of smoke (K)
*u*: smoke velocity (m/s)
*V*: subway speed (m/s)
*V_t_*: smoke longitudinal velocity (m/s)
*Y_M_*: fluctuating dilatation in turbulence to the overall dissipation rate
*Z*: height of smoke relative to tunnel ground (m)
Greek symbols *α_k_*: inverse effective Prandtl number of k equation
*αε*: inverse effective Prandtl number of ε equation
*μ_eff_*: effective viscosity coefficient
*ρ*: ambient density (kg/m3)
*ε*: turbulent dissipation rate
Subscript c: concentration of smoke in numerical simulation
max: maximum value of a parameter
Expt: value in the experiment of a parameter
Diff: difference between parameter values
